# Nonparametric methods for the analysis of single-color pathogen microarrays

**DOI:** 10.1186/1471-2105-11-354

**Published:** 2010-06-28

**Authors:** Omar J Jabado, Sean Conlan, Phenix-Lan Quan, Jeffrey Hui, Gustavo Palacios, Mady Hornig, Thomas Briese, W Ian Lipkin

**Affiliations:** 1 Center for Infection and Immunity Mailman School of Public Health Columbia University New York, NY USA

## Abstract

**Background:**

The analysis of oligonucleotide microarray data in pathogen surveillance and discovery is a challenging task. Target template concentration, nucleic acid integrity, and host nucleic acid composition can each have a profound effect on signal distribution. Exploratory analysis of fluorescent signal distribution in clinical samples has revealed deviations from normality, suggesting that distribution-free approaches should be applied.

**Results:**

Positive predictive value and false positive rates were examined to assess the utility of three well-established nonparametric methods for the analysis of viral array hybridization data: (1) Mann-Whitney *U*, (2) the Spearman correlation coefficient and (3) the chi-square test. Of the three tests, the chi-square proved most useful.

**Conclusions:**

The acceptance of microarray use for routine clinical diagnostics will require that the technology be accompanied by simple yet reliable analytic methods. We report that our implementation of the chi-square test yielded a combination of low false positive rates and a high degree of predictive accuracy.

## Background

Improvements in microarray fabrication and scanning technologies have enabled the production of high-density arrays that can facilitate the detection of all viral isolates, even those belonging to large, diverse families. Methods have been developed to identify conserved nucleic acid regions that are common to larger groupings (lineages, serogroups, genera, or families), thereby minimizing the number of probes required and increasing the chance of detecting novel viruses. The more recent strategies for achieving this include (i) using pairwise sequence comparisons to identify conserved sequences for probes [1-3] as well as (ii) identifying specific regions from a multiple sequence alignment [4-6]. Additionally, our group developed a method for designing probes within conserved protein regions [7]. Finally, oligonucleotide tiling arrays used in viral resequencing have been applied to both virus detection [8-11] and viral transcript profiling [12].

Although best practices have been established for fluorescent signal analysis in expression profiling [13], standardization has not yet been attained for pathogen detection arrays. While low-density microarrays can be analyzed by visual inspection [14], high-density arrays require computational solutions. Correlation of fluorescence to a predicted hybridization signal has been used to infer the presence of a virus [15], while a t-test based method has also been validated with the same datasets [16]. Other methods include semi-supervised classification using the K-nearest neighbor technique [17], an empirical determination of signal cutoffs for tiling arrays [18], and a likelihood metric informed by taxonomic hierarchies [19]. Resequencing arrays are able to leverage redundancy and deep-coverage, thereby helping the scientist to infer what the viral nucleotide sequence is [10], but whether highly divergent strains of viruses can be correctly annotated by this method is unclear.

Pathogen microarray data is noisy; viral signatures can be masked by cross-hybridizing host transcripts, biased template amplification, imperfectly matching probes or as the result of different probe sensitivities. Additionally, only a small proportion of probes are likely to hybridize if virus is present in a sample, leading to distributions with high variance. Nonparametric tests transform data into rank order or categories; this has the effect of rescaling variance and simplifying the distribution to one more easily modeled. If data variance is high or the distribution is heavily skewed, nonparametric tests are more conservative than equivalent parametric tests. What is more, nonparametric tests are valid with small sample sizes (less than 20); this is an advantage in cases where the presence of a virus is being investigated by a small number of probes. Nonparametric methods such as the Mann Whitney *U *test have been applied in detecting differential gene expression of by microarrays [20].

We compared the ability of three specific nonparametric statistical tests to predict the presence of viral agents in a hybridization experiment The three tests were (1) the Mann-Whitney *U*, a test of central tendency; (2) the Spearman rank correlation coefficient, a measure of the relation between two variables; and (3) the chi-square, a test of event probability based on the binomial distribution. Along with negative controls, nine viral isolates from different families were hybridized; the isolates had genome sizes ranging from 7 to 156 kilobases. Type I errors that can result from multiple testing were controlled by using the method created by J.D. Storey [21]. Positive predictive value and false positive rates were used to assess the ability of the three different statistical methods to identify viruses.

## Methods

### Reference array design and hybridization

Microarrays were fabricated wherein probes were synthesized *in situ *(at Agilent Technologies Inc., Santa Clara, CA) in two orientations: plus (coding sense) and minus (non-coding antisense, the reverse complement); all probes were covalently anchored at the 3' end [7]. For testing purposes, a microarray was fabricated wherein 8,553 probes were deposited in both orientations and in duplicate (GreeneChip Pilot: 38,546 total probes, barcode 25161471, Gene Expression Omnibus (GEO) Platform GPL10319, http://www.ncbi.nlm.nih.gov/geo). Another microarray was fabricated with a larger number of virus specific probes deposited in both orientations but singly (GreeneChip 1.52RC: 31,771 total probes, barcode 25177711, GEO Platform GPL10320). Negative control probes were generated from 1,500 randomly-chosen shuffled viral probe sequences. These probes used the same proportion of nucleotides as the viral probes, but were not homologous to any known sequences. All probes were randomly positioned on the array.

To approximate clinical sample contexts yet allow for examination of a wide range of viral targets, hybridization experiments were conducted in which known concentrations of WNV viral extract were spiked into reactions containing either 10 or 200 nanograms (ng) of human lung tissue RNA (data available in GEO Series GSE21317; see summary of data availability in additional file 1).

We employed viruses that varied in genome type and length to assess the sensitivity and specificity of three methods for statistically analyzing viral microarray data. The data pool included three single-stranded positive sense RNA viruses: West Nile virus (WNV, strain New York 1999, AF202541, ca 11 kb), SARS coronavirus (HCoV-SARS, strain Tor2, AY274119, ca 30 kb), and human echovirus 18 (EV18, strain Metcalf, ATCC VR-48, AF317694, ca 7.4 kb); two segmented single-stranded negative sense RNA viruses: Lassa virus (LASV, strain Josiah, ca 3.4 kb and 7.3 kb) and influenza A virus H1N1 (FLUA H1N1; A/Texas/36/91, CY009316-CY009323, 8 segments of ca 2.3, 2.3, 2.2, 1.7, 1.5, 1.4,1 and 0.9 kb); two non-segmented single-stranded negative sense RNA viruses: Zaire ebolavirus (ZEBOV, NC_002549, ca 19 kb) and vesicular stomatitis virus (VSV, Indiana strain, NC_001560, ca 11.1 kb); and two double-stranded DNA viruses: human adenovirus 4 (HAdV-4, ATCC VR-1572, NC_003266, ca 36 kb) and human herpesvirus 1 (HSV-1, viral culture, NC_001806, ca 152 kb) (see Table 1; data available in GEO Series GSE21318 and Additional File 2).

**Table 1:** Viral isolates

Genome Type	Approximate genome size (kb)	Family	Virus/strain	Abbreviation
+ sense ssRNA	11	Flaviviridae	West Nile virus New York 1999	WNV
+ sense ssRNA	30	Coronaviridae	SARS Human Coronavirus Tor2	HCoV-SARS
+ sense ssRNA	7.4	Picornaviridae	Human Echovirus 18 Metcalf	EV18
- sense ssRNA	3.4 and 7.3	Arenaviridae	Lassa virus strain Josiah	LASV
- sense ssRNA	19	Filoviridae	Zaire ebolavirus	ZEBOV
- sense ssRNA	11	Rhabdoviridae	Vesicular stomatitis virus Indiana	VSV
- sense ssRNA	0.89 to 2.3	Orthomyxoviridae	Influenza A H1N1 Texas 1991	FLUA H1N1
dsDNA	36	Adenoviridae	Human adenovirus 4	HAdV-4
dsDNA	156	Herpesviridae	Human herpesvirus 1	HSV-1

After both random amplification and dendrimer labeling [22,23] were completed, the arrays were visualized with an Agilent slide scanner. SPSS version 16 http://www.spss.com was used for statistical analysis and data plotting.

### Computation of probe-target characteristics

A database of probe-virus target homologies was created for use in our analysis algorithms. The EMBL viral nucleotide sequence database [24] was filtered for short HIV-1 sequences and combined with the NCBI viral reference-sequence database [25]. A non-redundant database of 74,044 sequences was generated with CD-Hit [26], using a similarity cutoff of 98% to define sequences as identical. All probe sequences were compared to the non-redundant set of viral sequences; the number and position of any mismatches was stored for each probe-virus target pair. Change in Gibbs free energy (ΔG) at 65°C (hybridization temperature) was calculated as a measure of probe-target binding strength [7], a table of values is available in GEO Series GSE21319. The ΔG of probe-reverse complement hybrids was also calculated; probes with higher GC content had a greater ΔG. A Lowess fit of probe-reverse complement ΔG and the fluorescent signal was computed for all hybridizations and used to correct for sequence composition [27].

Closely related viruses may have indistinguishable hybridization profiles (e.g., the intensity of signal from probes will have the same values). We identified a set of viral sequence records likely to be distinguishable by the array. Using the ΔG values, we computed an *in silico *hybridization profile and used it to compute distances [28]. A cut-off of -32 kJ was used to determine whether a probe was likely to hybridize. *In silico *hybridization profiles that differed by less than 6 probes were consolidated.

### Cross validation with a public dataset

To assess the applicability of our analytic method to another pathogen microarray platform, we used the E-Predict training set [15] (http://www.ncbi.nlm.nih.gov/geo/ accessions GSM40806 to GSM40861 inclusive). The dataset was comprised of 56 ViroChip microarray hybridizations of viral samples previously characterized by direct immunofluorescence. The viruses represented were human papillomavirus 18 (15 samples derived from HeLa cells), influenza A virus (8 samples derived from nasal lavage), hepatitis B (1 sample derived from serum), respiratory syncytial virus (10 samples derived from nasal lavage) and human rhinovirus (22 samples derived from viral culture in cell lines). One clinical isolate of influenza A virus also contained a respiratory syncytial virus (GSM40845). The array was comprised of 12,505 probes; the 265 probes excluded from the E-Predict analysis were also excluded from this study.

### Statistical tests and implementation

Three well-known statistical tests were used to evaluate the presence or absence of viruses in our hybridization experiments (see Additional File 3, Figure S1). A set of negative control probes was used as a reference. Probe pairs for both the plus (coding sense) and minus (non-coding antisense) orientations were present on the array. We assessed the relative utility of probe pairings by performing statistical tests using the plus strand alone and the minus strand alone, and by pooling the pairs together. Permutation tests were performed when needed to determine significance. Programs for statistical computation were written either in Perl or with modules from the R Project for statistical computing http://www.r-project.org.

#### Mann-Whitney *U *Test

T-tests and ANOVA tests are the most common form of statistical tests in microarray analysis literature [29], and have been successfully applied to pathogen arrays [16]. The Mann-Whitney *U *test (also called the "Wilcoxon rank-sum test") is the nonparametric analogue of the Student's t-test. By using ranked data, it assesses whether two groups of samples are drawn from the same distribution.

In our experimental application, the signals of viral probes that targeted a single strain were compared to an equal number of negative control probes. If a virus was targeted by less than 10 probes, negative control probes were supplemented to achieve an *n *of 20. The null hypothesis (H_0_) was that virus specific probes and negative control probes had the same central tendency (e.g., they were drawn from the same population). In each instance, the significance of the *U *statistic was computed with the commonly-used normal approximation for large samples [30].

#### Spearman correlation

Correlation is the strength of the linear relationship between two variables. The most common method of assessing correlation between two continuous variables is Pearson's product-moment correlation coefficient. A normal data distribution is not a requirement of the test although equality of variance (or "homoscedasticity") is assumed [31]. The variance assumption has been shown to be a confounder in microarray analysis [32]; thus we decided to use a more conservative correlation measure that makes use of ranked data: namely, Spearman's rank correlation coefficient.

In this application, the change in the Gibbs free energy values of the predicted probe-virus target hybrids was used as the independent variable, while the dependent variable was the observed probe signal (see [7] for a graphical example). ΔG was used to predict fluorescence and to model surface hybridization kinetics [33,34]. An equal number of negative control probes were pooled with viral probes to ensure that a wide range of signals would be examined. This was necessary to ensure that a correlation could be computed, because a uniformly high signal would be regarded as uncorrelated to the ΔG value. For negative control probes, the independent variable was the ΔG of the predicted human ribosomal RNA duplexes. The null hypothesis was that a fluorescent signal would not be predicted by ΔG (e.g., the signal might, rather, be randomly distributed with respect to ΔG). One sided p-values for correlations were computed using a standard Student's t-distribution approximation [30].

#### Binomial Test

The binomial test is a subset of the *X*^*2 *^(chi-square) method; it tests the probability of observing a series of events according to an expected probability rate. An example of this kind of test would be an assessment of the fairness of gaming dice [30].

In this application, we "binned" the probe signals into percentiles and then categorized them as positive or negative based on whether their computed values were located above or below a certain threshold. Probes for a single virus were tested against the *X*^*2 *^distribution in such a way that the expected probability was equal to the probability rate of the negative control probes. The null hypothesis was that above the threshold, there was no difference between the proportion of virus specific probes and negative control probes. The threshold choice for the binomial test clearly has a strong influence on the results; our own exploratory testing of various percentile thresholds (70^th^, 80^th^, 90^th^, 95^th ^and 99^th^) showed that the 90^th ^percentile generated the best performance for our needs. All binomial tests were conducted using the 90^th ^percentile in categorizing probes as "positive" or "negative." We concluded that the threshold value for the test, whatever it might be, should be considered a parameter for optimization.

### Multiple test correction via the False Discovery Rate (FDR)

An issue that often arises in testing a series of null hypotheses is the increase in the probability of the occurrence of a type I error (e.g., the rejection of H_0 _when it is true). Conservative familywise correction methods such as Bonferroni's have been applied to microarray data. An alternative method, first described by Benjamini and Hochberg [35] and then revised by Storey [36], involves correcting the proportion of falsely rejected hypotheses. Here we used Storey's method and defined its parameters, within our study, as the proportion of the viruses identified through array hybridization that prove to be false leads (i.e., the q-value) [21]. In a multiple hypothesis test scenario, requiring a q value of ≤ 5% for there to be "significance" would indicate that up to 5% of the viruses identified as present in a sample may, in fact, have been truly absent. In contrast, requiring a p value of ≤ 5% for there to be "significance" indicates that up to 5% of all truly absent viruses may be identified as present. Q-values were calculated from test p-values by using the *q-value *module from the R Project contributed by Storey.

### Assessment of test performance

A table of correct predictions for each hybridized virus was created by using the NCBI Taxonomy database (available as Additional File 4). A prediction was considered a "true positive" if either the specific virus introduced in the experiment or any other virus in the same genus was, thereby, predicted. Q-values with a threshold of 10% were used; and the top 250 predictions for each method were evaluated. Species prediction could also have been assessed, but would have been less indicative of algorithm performance because the concept of what constitutes a viral species is not comparable between the various taxonomic families (e.g., by serology or by molecular phylogeny). What is more, highly similar strains may be impossible to distinguish by examining their microarray signature, yet still be defined as different species; in contrast, viruses from different genera are more easily distinguishable.

False positive rate (FPR) and positive predictive value (PPV) were used to assess the performance of the various test models. PPV is a commonly used standard for diagnostic tests; it is the probability that a positive test result will reflect the presence of a virus in the sample. PPV is the ratio of the number of true positives to the sum of the number of true positives and false positives; false negatives are not used in the PPV method. In our particular application of the method, it was the ratio of the number of statistically significant (*q *≤ 0.10) and correct predictions to the sum of all the statistically significant predictions generated; predictions below the significance threshold were ignored. PPV is a useful measure of algorithm performance because it is not sensitive to heterogeneity or misclassification in viral taxonomy; it is not necessary to predict that all members of a genus are present in order to achieve a favorable score.

## Results

### Deviation from normality in fluorescence distribution

**Figure 1:**
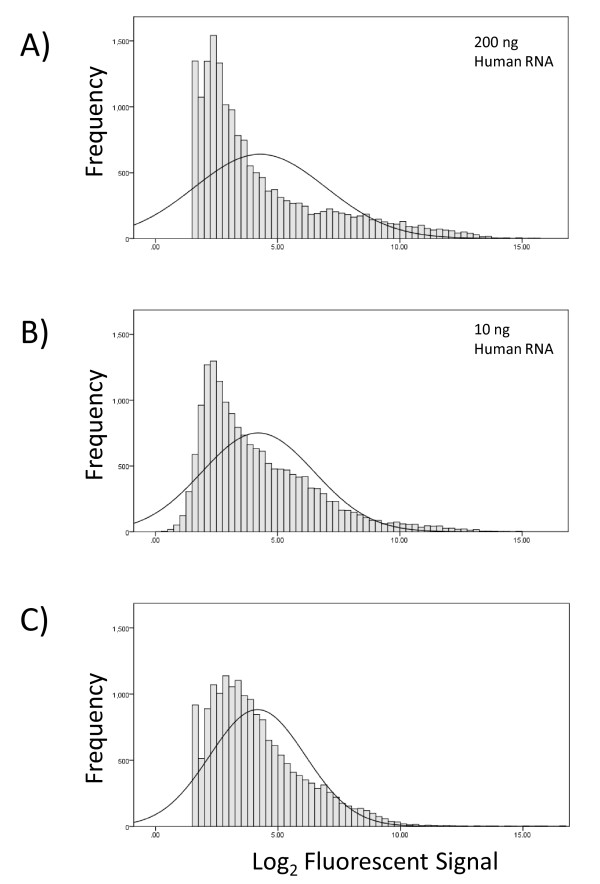
**The effect of increasing human nucleic acid on the hybridization signal**. Three quantities, 200, 10 and 0 ng of human lung RNA (panels a, b, c), were hybridized to a pathogen microarray comprised of ~38,000 probes. The fluorescent signal from the pathogen-specific probes was log-transformed and used to generate histograms. An expected normal (Gaussian) distribution is depicted by the line in the above figure. Tests for normality indicated significant deviation, with the following Kolmogorov-Smirnov Z values: (a) 41.5, (b) 14.6, (c) 12.1 (all *p *< 0.01).

To assess the effects of variability that derive from the concentration of viral nucleic acid and host nucleic acid in clinical samples, we characterized the effect of a varying input of human RNA on the fluorescence distribution of the log-transformed probe signals. While an analysis by Giles and Kipling of Affymetrix arrays based on 25 nt probes indicated that the fluorescence distributions of their log-transformed probe signals were indeed close to normal (Gaussian) [37], our experience with 60 nt probe arrays showed strong deviations from a normal distribution. Increases in RNA input have complex effects on signal distribution: the degree of skew is increased and the upper tail is lengthened (see Figure 1). We tested the normality of these distributions and found strong deviations for all three experiments (using the Kolmogorov-Smirnov test, where p < 0.05). Sample distributions of fluorescent signals deriving from virus-infected tissue-culture cell hybridizations are available in Additional file 5, Figure S2. The observed signal distributions included Gaussian, highly kurtotic and bimodal; host RNA concentration can only partially explain these differences. Other explanations include: a large proportion homologous probes on the array increasing the upper tail, differences in amplification or labeling efficiency from sample to sample, and differences in hybridization affinity due to guanine/cytosine proportion in viral transcripts. While more complex methods of transformation have been described elsewhere, these have focused on improving the sensitivity of transcript profiling measurements and, we surmised, were unlikely to improve sensitivities in our application. Parametric analysis of microarray data is particularly sensitive to transformations; violations of normality will lead to a loss of power, and may result in invalid p values [38].

### Reverse complement controls reduce noise

Replication of hybridization experiments can improve specificity [39]; however, the expense incurred by processing multiple arrays or low quantities of nucleic acid in one's clinical sample may preclude the use of this approach. Alternative strategies that might be adopted include the application of either replicate probes and/or reverse complementary probes to the same array. A study using Agilent 60 nt *in-situ *synthesized arrays found that replicate probes were highly concordant [40], and useful both in improving normalization and in identifying outlier signals [41]. Reverse complements have been used on spotted oligonucleotide arrays for pathogens [1], although no quantitative assessments of their advantages have been reported up to this point. In such studies as are mentioned above, *in situ *synthesized oligonucleotides are immobilized at the 3' end. Probe-target helix stability near the surface is likely to be reduced due to steric hindrance. Thus, probes that are mismatched to their targets will be strongly affected by the specific mismatch position (e.g., mismatches near the 5' end will reduce the hybridization signal more than those near the 3' end [42]).

To identify a printing strategy that would reduce false positive signals, we conducted pilot studies using infected cell extracts of WNV containing 10^4 ^and 10^6 ^copies of viral template RNA as input for the reverse transcriptase reaction. Amplification products were hybridized on an array containing both replicate and reverse complement probes, while human lung RNA was used as a control. In all experiments, replicate probe pairs were better correlated than reverse complement pairs for both WNV cognate probes and non-flavivirus probes. When WNV was present, reverse complement probes had a higher correlation than when no WNV was present; this differential response was stronger in probes that had the highest degree of signal (i.e., those in the top 10^th ^percentile) (see Table 2 and Additional File 6, Figure S3).

**Table 2:** Correlation between replicate and reverse complemented probe pairs

Pearson Correlation (R)		**10**^**6 **^**copies input**		**10**^**4 **^**copies input**		No West Nile Virus	
		
		Replicates	Reverse Complements	Replicates	Reverse Complements	Replicates	Reverse Complements
Overall	West Nile Virus probes	0.92	0.85	0.88	0.72	0.83	0.27
	non-target probes	0.72	0.27	0.75	0.31	0.79	0.35

Tenth percentile	West Nile Virus probes	0.90	0.83	0.84	0.68	0.77	·-0.01 †
	non-target probes	0.86	0.36	0.85	0.40	0.79	0.19

		n = 11		n = 12		n = 3	

*p < .05 *for all except †

### Correcting for sequence composition reduces the false positive rate

Seven negative control hybridizations and nine viral isolate hybridizations were used to compute false positive rates (FPR) (see Table 1). The top 250 predictions for each statistical test were evaluated. A q-value threshold of 10% was applied; this corresponds to a condition in which 1 in 10 predictions may prove to be false. The binomial test performed well, achieving a 2% false positive rate, whereas the Mann-Whitney *U *and Spearman correlation tests had high average FPRs (48% and 58% respectively; see Table 3). We noticed that a strong relationship existed between the GC content and the fluorescent signal in our platform (see Additional file 7, Figure S4); it is worth noting that high GC content h	as previously been implicated in off-target hybridization [43,44]. We applied a probe-level correction and found that a substantial decrease in the FPR occurred in the case of both the Mann-Whitney *U *and Spearman correlation methods as a result (see Table 3; data available in additional file 8 and additional file 9).

**Table 3:** False positive rates for methods of pathogen identification

	False Positive Rate					
	
Target	Mann-Whitney *U*		Spearman Correlation		Binomial test	
		GC Corrected		GC Corrected		GC Corrected
WNV	0%	0%	0%	0%	0%	0%
HCoV-SARS	7%	0%	100%	0%	0%	0%
EV18	63%	15%	100%	12%	14%	0%
LASV	11%	0%	100%	0%	0%	0%
ZEBOV	0%	0%	0%	0%	0%	0%
VSV	0%	0%	0%	0%	0%	0%
HAdV-4	100%	30%	100%	5%	18%	0%
FLUA H1N1	20%	1%	100%	0%	0%	0%
HSV-1	8%	0%	0%	1%	2%	0%
Negative Control (200 ng HuDNA)	100%	0%	1%	0%	1%	0% †
Negative Control (200 ng HuDNA)	100%	0%	100%	1%	1%	0%
Negative Control (200 ng HuDNA)	100%	0%	100%	1%	1%	0%
Negative Control (200 ng HuDNA)	100%	0%	100%	0%	1%	0%
Negative Control (10 ng HuDNA)	100%	0%	88%	0%	2%	0%
Negative Control	100%	0%	100%	0%	0%	0% †
Negative Control	0%	0%	0%	0%	0%	0%
Negative Control	4%	0%	2%	0%	0%	0%

**Average**	**48%**	**3%**	**58%**	**1%**	**2%**	**0%**

† Hybridization performed on different days

Even after GC correction, a strong prediction for a chicken endogenous retrovirus was identified from the influenza virus hybridization by all three methods. The virus was cultured in eggs that are likely to express the viral transcripts, indicating that a microarray can indeed identify co-infections.

### A binomial test correctly predicts the viral genus in a majority of cases

Statistical methods were compared for GC-corrected signals by using PPV (Positive Predictive Value), which is the probability that a diagnostic test will correctly report the causative agent of an illness. In clinical applications, the identification of a viral genus is sufficient to direct other molecular identification methods (e.g., consensus PCR) as well as to prescribe initial clinical measures. Thus, we measured the success of each statistical method by whether it correctly predicted the presence of a hybridized viral isolate or any other virus within the same genus. The precise number of times that the most statistically significant prediction proved to be correct was also tabulated (see Table 4 for an enumeration of both measures). Statistical analysis was carried out with coding sense probes, antisense probes, or both probe types pooled together, as independent measures of the quantity of viral nucleic acid. A strategy of averaging probe values resulted in a performance similar to that of those instances when coding sense probes alone were used.

**Table 4:** Positive predictive value for methods of pathogen identification

	**Positive Predictive Value**								
**Target**	**Mann-Whitney *U***			**Spearman Correlation**			**Binomial test**		
	**sense**	**anti-sense**	**both**	**sense**	**anti-sense**	**both**	**sense**	**anti-sense**	**both**
WNV	100%	100%	100%	0%	0%	0%	100%	100%	100%
HCoV-SARS	50%	33%	50%	0%	0%	0%	50%	100%	100%
EV18	92%	95%	91%	92%	100%	96%	95%	100%	96%
LASV	0%	0%	0%	0%	0%	0%	0%	0%	100%†
ZEBOV	100%	100%	100%	0%	0%	0%	100%	100%	100%
VSV	100%	100%	100%	0%	0%	0%	100%	100%	100%
HAdV-4	48%	74%	32%	0%	100%	0%	85%	89%	73%
FLUA H1N1	99%	96%	95%	100%	100%	100%	100%	100%	97%
HSV-1	83%	100%	71%	0%	0%	0%	63%	100%	54%
**Average**	**75%**	**77%**	**71%**	**21%**	**33%**	**22%**	**77%**	**88%**	**91%**
**Correct top ranked predictions**	**8/9**	**8/9**	**8/9**	**2/9**	**3/9**	**2/9**	**8/9**	**8/9**	**9/9**

Top 250 predictions; *q value *threshold = 0.1

† LASV is targeted by four probes

The binomial test obtained the highest PPV of 91% when both sense probes and antisense probes were taken into account, while the Mann-Whitney *U *test also performed favorably. In contrast, the Spearman correlation coefficient test had a substantially lower PPV of 33%. A relaxation of the multiple-testing correction requirements resulted in an improvement in the Spearman PPV for antisense probes to 76% but such a relaxation of correction requirements is undesirable due to the potential rise in false positives that might result (see Additional file 10, Table S1). In two cases (those of HSV-1 and HAdV-4), the use of both sense and antisense probes reduced the overall PPV; however the input virus was nonetheless correctly predicted. In another case (that of the LASV, binomial test), the pooling of the two probe methods resulted in detection where the virus would otherwise have been missed.

### Validation of binomial and Mann-Whitney *U *test performance on the ViroChip platform

A publically available dataset from Urisman et al. (ViroChip E-Predict training set; [15]) was downloaded and analyzed using the Mann-Whitney *U *and binomial tests. The ViroChip dataset was comprised of 56 hybridizations of samples derived from tissue culture, nasal lavage or serum. The samples contained papillomavirus, influenza A virus, hepatitis B virus, respiratory syncytial virus or human rhinovirus. The ViroChip platform differs from ours in the following ways: oligonucleotide probe length (70 nt vs. 60nt), number of probes (~12,000 vs. ~30,000), fabrication method (robot spotted vs. *in situ *synthesis), incorporation of fluorescence (amino-allyl dUTP vs. secondary hybridization) and presence of GC-matched negative control probes. However, the ViroChip platform does include the use of reverse complement probes and employs a random-PCR based nucleic acid amplification strategy that is similar to ours.

Two other methods (E-Predict [15] and DetectiV [16]) reported a high degree of predictive accuracy when they were used to examine the same dataset. Similarly, we report having achieved a high degree of accuracy when using two nonparametric testing methods (binomial test and Mann-Whitney *U*) together with the same parameters that were used for our *in-situ *synthesized probe platform. We report on our performance in this instance by relying on a simple metric: the rank of the first correct prediction (see Table 5). The first prediction of the Mann-Whitney *U *test was correct in 47% of the cases; a correct prediction was present among the first ten predictions in 91% of the cases. The average correct prediction rank for the Mann-Whitney *U *test was 3.7. The largest number of incorrect predictions were for human herpesvirus 7 (HHV7), human herpesvirus 6 (HHV6A/B) and human herpesvirus 5 (HHV5), regardless of the sample source. The first prediction of the binomial test was correct in 75% of the cases; a correct prediction was present among the first ten predictions in 98% of the cases. The average correct prediction rank for the binomial test was 1.6. As in the Mann-Whitney *U *results, the majority of incorrect predictions were for HHV7. Watson et al. reported a similar false-positive result using a t-test method [16]. HHV7 has a seroprevelance of 85% in humans and can be readily detected in blood, saliva and cervical tissue [45]. The degree of prevalence suggests that the predictions represent a true co-infection; however, validation of this would require further molecular tests. Both the binomial and Mann-Whitney *U *methods generated statistically significant predictions for an influenza virus and RSV co-infected sample.

**Table 5:** Performance of Mann-Whitney U and Binomial tests with ViroChip pathogen microarray platform

	Respiratory Syncytial virus		Rhinovirus		Influenza A		Papillomavirus		Hepatitis B		Total	
Rank of First Correct Prediction	Mann Whitney U	Binomial Test	Mann Whitney U	Binomial Test	Mann Whitney U	Binomial Test	Mann Whitney U	Binomial Test	Mann Whitney U	Binomial Test	Mann Whitney U	Binomial Test
First	40%	50%	77%	86%	29%	71%	20%	80%	0%	0%	47%	75%
Top 5	90%	100%	100%	95%	43%	86%	67%	100%	100%	100%	82%	96%
Top 10	100%	100%	100%	95%	57%	100%	87%	100%	100%	100%	91%	98%

Average Rank	2.5	1.7	1.2	1.6	10.7	2.3	4.7	1.3	4	2	3.7	1.6
Experiments	10	10	22	22	7	7	15	15	1	1	55	55

The binominal test outperformed the Mann-Whitney test on the ViroChip data, largely because Mann-Whitney's ranking on HHV7 was lower. The binomial test's performance without optimization was comparable to that of E-Predict (95% correct for first predictions, 100% correct for the first ten) and to that of DetectiV (98% correct for first predictions, 100% correct for the first ten), however, a binomial test does not require probe-level weighting or assumption of normality.

## Discussion

Although expression profiling and viral microarrays may employ similar methods for printing probes and for preparing and hybridizing nucleic acids, they differ markedly in their analytical strategy. A fundamental assumption in expression profiling is that the majority of the various gene transcript mRNAs are present in the sample (albeit at different concentrations). Differential expression is identified by computing the ratio of probe signal for a gene in two conditions (e.g., tumor vs. normal tissue). In a viral microarray experiment, absence of signal from all probes is a plausible result. This may reflect any of the following: (1) that no agent is present; (2) that an agent is present but there are no probes for it because it is either truly new or sufficiently different to confound hybridization; (3) that a known agent is present and that probes are appropriate but that levels are insufficient to enable detection.

The results presented here indicate that the quantity of host nucleic acid used in hybridization has substantive effects on a probe's fluorescent signal distribution that manifest as deviations from normality. We speculate that these deviations represent mass effects when concentrations are high enough to drive up the proportion of partially hybridized strands. Probe sequence composition was identified as a major confounder that resulted in high FPR. Probe signal correction by GC content improved predictions. We tested the effect of various probe control strategies on the FPR. A hybridization requirement for both sense and antisense probes was more helpful in reducing the FPR than the use of hybridization to replicate probes. An additional important negative control was the inclusion of shuffled viral probes that enabled examination of the effects of array-wide GC content.

A key strength of this study was its effective control of the familywise error rate, achieved via its use of Storey's FDR technique [36]. While this method successfully lowered the FPR, there was a mild violation in the independence assumption as some probes target multiple viruses. Q values remain useful when p values are correlated within blocks [21], a condition we expect to be true within our probe-sets. Methods that explicitly address dependence have recently been under investigation [46] and should be incorporated in any future versions of our method.

Nonparametric statistical methods are more conservative than equivalent parametric ones; consequently, they are less likely to require that one reject the null hypothesis when it is false (as in the case of type II errors or false negatives). The variability of microarray signal distributions in our study suggested that parametric predictions may be inaccurate; for this reason, we tested a number of nonparametric approaches. Of the three methods that we assessed, the binomial test was the most successful, achieving a 91% PPV. A further development of our study might include empirically determining a more optimal threshold by using spike-in controls or by adding more categories to reflect different levels of confidence (e.g., positive, marginal and negative). The Mann-Whitney *U *test was also successful in that it predicted the presence of nearly all agents. This result conforms with a similar study using t-tests [16]. Yet the Mann-Whitney *U *assessment was sensitive to sequence composition; its PPV on uncorrected signal data was only 48%.

The Spearman correlation performed poorly, correctly predicting only three disease agents. A Pearson correlation method (E-Predict) has been successfully applied to pathogen arrays, according to Urisman et al. [15]. The E-predict method performs an *in silico *hybridization between fully sequenced viral genomes and a viral array. After a clinical sample is hybridized, the predicted fluorescence for each virus is compared to the observed signal. The strength of correlation is used to identify which virus, if any, is responsible for the signal pattern. In our study and in Urisman's, the Spearman correlation performed poorly. We re-explored this result by relaxing the requirement for a multiple testing correction and found that it improved the degree of predictive accuracy, but the Spearman assessment still did not perform as well as the other tests (see Additional file 10, Table S1). Accordingly, we concluded that the Spearman correlation as implemented was not as discriminative as other nonparametric tests.

## Conclusion

We report the successful application of two nonparametric tests that require few assumptions that have a high degree of predictive accuracy and have extremely low false positive rates. In a direct assessment of the binomial and Mann-Whitney *U *tests on a related pathogen microarray platform, the binomial test performed comparably to other reported methods (e.g., E-Predict [15] and DetectiV [16]). In contrast to these methods, the binomial test achieved a high degree of predictive accuracy on ranked data without probe-specific weighting parameters, iterative analysis or assumptions of normality.

## Authors' contributions

PQ and JH carried out the virus culture, quantitation and microarray hybridization experiments. GP and TB helped to design microarrays and devise experiments. MH provided statistical advice. OJ and SC programmed the analytic technique and wrote the manuscript. WIL and OJ designed and analyzed the various experiments. All authors read and approved the final manuscript.

## Supplementary Material

Additional File 1 **Hybridization summary**. Summary of viral and control hybridizations, corresponding GEO accession numbers and location of data presented in manuscript. Click here for file

Additional File 2 **Fluorescent signal data used in table 1**. Compressed file in CSV format containing a table of signal values where probe reverse complements and replicates are in rows. Click here for file

Additional File 3 **Figure S1**. Graphical description of non-parametric tests evaluated in study Click here for file

Additional File 4 **Viral genus classification table**. File contains a table of viral accession numbers in rows, and a list of viruses in columns. Values are a boolean for whether the viral sequence in the row is considered to be in the genus of the virus listed at the top of the column (1 = in the genus). This table was used to calculate positive predictive value. Click here for file

Additional File 5 **Figure S2**. Histograms of fluorescent signal for hybridizations to a pan-viral microarray. Click here for file

Additional File 6 **Figure S3**. Comparison of probe printing strategies for pathogen arrays. Click here for file

Additional File 7 **Figure S4**. Relationship between fluorescence and probe sequence composition. Click here for file

Additional File 8 **Fluorescent signal data used in table 3**. Compressed file in CSV format containing a table of signal values where probe reverse complements and replicates are in rows. Data is not corrected for probe sequence composition. Click here for file

Additional File 9 **Fluorescent signal data used in table 3**. Compressed file in CSV format containing a table of signal values where probe reverse complements and replicates are in rows. Data is corrected for probe sequence composition using a Lowess fit. Click here for file

Additional File 10 **Table S1**. Positive predictive value for methods of pathogen identification with no multiple testing correction. Click here for file

## References

[B1] Wang D Coscoy L Zylberberg M Avila PC Boushey HA Ganem D DeRisi JL Microarray-based detection and genotyping of viral pathogens Proc Natl Acad Sci USA 2002 99 24 15687 15692 10.1073/pnas.242579699 12429852 PMC137777

[B2] Lin FM Huang HD Chang YC Tsou AP Chan PL Wu LC Tsai MF Horng JT Database to dynamically aid probe design for virus identification IEEE Trans Inf Technol Biomed 2006 10 4 705 713 10.1109/TITB.2006.874202 17044404

[B3] Chou CC Lee TT Chen CH Hsiao HY Lin YL Ho MS Yang PC Peck K Design of microarray probes for virus identification and detection of emerging viruses at the genus level BMC Bioinformatics 2006 7 232 10.1186/1471-2105-7-232 16643672 PMC1523220

[B4] Chizhikov V Wagner M Ivshina A Hoshino Y Kapikian AZ Chumakov K Detection and genotyping of human group A rotaviruses by oligonucleotide microarray hybridization J Clin Microbiol 2002 40 7 2398 2407 10.1128/JCM.40.7.2398-2407.2002 12089254 PMC120567

[B5] Laassri M Chizhikov V Mikheev M Shchelkunov S Chumakov K Detection and discrimination of orthopoxviruses using microarrays of immobilized oligonucleotides J Virol Methods 2003 112 1-2 67 78 10.1016/S0166-0934(03)00193-9 12951214 PMC9533938

[B6] Mehlmann M Dawson ED Townsend MB Smagala JA Moore CL Smith CB Cox NJ Kuchta RD Rowlen KL Robust sequence selection method used to develop the FluChip diagnostic microarray for influenza virus J Clin Microbiol 2006 44 8 2857 2862 10.1128/JCM.00135-06 16891503 PMC1594657

[B7] Jabado OJ Liu Y Conlan S Quan PL Hegyi H Lussier Y Briese T Palacios G Lipkin WI Comprehensive viral oligonucleotide probe design using conserved protein regions Nucleic Acids Res 2008 36 1 e3 10.1093/nar/gkm1106 18079152 PMC2248741

[B8] Wilson WJ Strout CL DeSantis TZ Stilwell JL Carrano AV Andersen GL Sequence-specific identification of 18 pathogenic microorganisms using microarray technology Mol Cell Probes 2002 16 2 119 127 10.1006/mcpr.2001.0397 12030762

[B9] Wong CW Albert TJ Vega VB Norton JE Cutler DJ Richmond TA Stanton LW Liu ET Miller LD Tracking the evolution of the SARS coronavirus using high-throughput, high-density resequencing arrays Genome Res 2004 14 3 398 405 10.1101/gr.2141004 14993206 PMC353227

[B10] Lin B Wang Z Vora GJ Thornton JA Schnur JM Thach DC Blaney KM Ligler AG Malanoski AP Santiago J Broad-spectrum respiratory tract pathogen identification using resequencing DNA microarrays Genome Res 2006 16 4 527 535 10.1101/gr.4337206 16481660 PMC1457032

[B11] Sulaiman IM Tang K Osborne J Sammons S Wohlhueter RM GeneChip resequencing of the smallpox virus genome can identify novel strains: a biodefense application J Clin Microbiol 2007 45 2 358 363 10.1128/JCM.01848-06 17182757 PMC1829075

[B12] Assarsson E Greenbaum JA Sundstrom M Schaffer L Hammond JA Pasquetto V Oseroff C Hendrickson RC Lefkowitz EJ Tscharke DC Kinetic analysis of a complete poxvirus transcriptome reveals an immediate-early class of genes Proc Natl Acad Sci USA 2008 105 6 2140 2145 10.1073/pnas.0711573105 18245380 PMC2542872

[B13] Shi L Reid LH Jones WD Shippy R Warrington JA Baker SC Collins PJ de Longueville F Kawasaki ES Lee KY The MicroArray Quality Control (MAQC) project shows inter- and intraplatform reproducibility of gene expression measurements Nat Biotechnol 2006 24 9 1151 1161 10.1038/nbt1239 16964229 PMC3272078

[B14] Townsend MB Dawson ED Mehlmann M Smagala JA Dankbar DM Moore CL Smith CB Cox NJ Kuchta RD Rowlen KL Experimental evaluation of the FluChip diagnostic microarray for influenza virus surveillance J Clin Microbiol 2006 44 8 2863 2871 10.1128/JCM.00134-06 16891504 PMC1594652

[B15] Urisman A Fischer KF Chiu CY Kistler AL Beck S Wang D DeRisi JL E-Predict: a computational strategy for species identification based on observed DNA microarray hybridization patterns Genome Biol 2005 6 9 R78 10.1186/gb-2005-6-9-r78 16168085 PMC1242213

[B16] Watson M Dukes J Abu-Median AB King DP Britton P DetectiV: visualization, normalization and significance testing for pathogen-detection microarray data Genome Biol 2007 8 9 R190 10.1186/gb-2007-8-9-r190 17868443 PMC2375028

[B17] Wiesinger-Mayr H Vierlinger K Pichler R Kriegner A Hirschl AM Presterl E Bodrossy L Noehammer C Identification of human pathogens isolated from blood using microarray hybridisation and signal pattern recognition BMC Microbiol 2007 7 78 10.1186/1471-2180-7-78 17697354 PMC1994958

[B18] Wong CW Heng CL Wan Yee L Soh SW Kartasasmita CB Simoes EA Hibberd ML Sung WK Miller LD Optimization and clinical validation of a pathogen detection microarray Genome Biol 2007 8 5 R93 10.1186/gb-2007-8-5-r93 17531104 PMC1929155

[B19] Rehrauer H Schonmann S Eberl L Schlapbach R PhyloDetect: a likelihood-based strategy for detecting microorganisms with diagnostic microarrays Bioinformatics 2008 24 16 i83 89 10.1093/bioinformatics/btn269 18689845

[B20] Troyanskaya OG Garber ME Brown PO Botstein D Altman RB Nonparametric methods for identifying differentially expressed genes in microarray data Bioinformatics 2002 18 11 1454 1461 10.1093/bioinformatics/18.11.1454 12424116

[B21] Storey JD Tibshirani R Statistical significance for genomewide studies Proceedings of the National Academy of Sciences of the United States of America 2003 100 16 9440 9445 10.1073/pnas.1530509100 12883005 PMC170937

[B22] Palacios G Quan PL Jabado OJ Conlan S Hirschberg DL Liu Y Zhai J Renwick N Hui J Hegyi H Panmicrobial oligonucleotide array for diagnosis of infectious diseases Emerg Infect Dis 2007 13 1 73 81 10.3201/eid1301.060837 17370518 PMC2725825

[B23] Quan PL Palacios G Jabado OJ Conlan S Hirschberg DL Pozo F Jack PJ Cisterna D Renwick N Hui J Detection of Respiratory Viruses and Subtype Identification of Influenza A Viruses by GreeneChipResp Oligonucleotide Microarray J Clin Microbiol 2007 45 8 2359 2364 10.1128/JCM.00737-07 17553978 PMC1951265

[B24] Cochrane G Aldebert P Althorpe N Andersson M Baker W Baldwin A Bates K Bhattacharyya S Browne P van den Broek A EMBL Nucleotide Sequence Database: developments in 2005 Nucleic Acids Res 2006 34 Database D10 15 10.1093/nar/gkj130 16381823 PMC1347492

[B25] Bao Y Federhen S Leipe D Pham V Resenchuk S Rozanov M Tatusov R Tatusova T National center for biotechnology information viral genomes project J Virol 2004 78 14 7291 7298 10.1128/JVI.78.14.7291-7298.2004 15220402 PMC434121

[B26] Li W Godzik A Cd-hit: a fast program for clustering and comparing large sets of protein or nucleotide sequences Bioinformatics 2006 22 13 1658 1659 10.1093/bioinformatics/btl158 16731699

[B27] Berger JA Hautaniemi S Jarvinen AK Edgren H Mitra SK Astola J Optimized LOWESS normalization parameter selection for DNA microarray data BMC Bioinformatics 2004 5 194 10.1186/1471-2105-5-194 15588297 PMC539276

[B28] Rash S Gusfield D String barcoding: uncovering optimal virus signatures RECOMB '02: Proceedings of the sixth annual international conference on Computational biology: April 18-21, 2002 2002 2002 Washington, DC, USA: ACM Press, New York, NY 254 261 full_text

[B29] Jafari P Azuaje F An assessment of recently published gene expression data analyses: reporting experimental design and statistical factors BMC Med Inform Decis Mak 2006 6 27 10.1186/1472-6947-6-27 16790051 PMC1523197

[B30] Daniel WW Biostatistics, a foundation for analysis in the health sciences 1987 4 New York: Wiley

[B31] Nefzger MD Drasgow J The Needless Assumption of Normality in Pearson-Tau American Psychologist 1957 12 5 623 625 10.1037/h0048216

[B32] Kristiansson E Sjogren A Rudemo M Nerman O Quality optimised analysis of general paired microarray experiments Stat Appl Genet Mol Biol 2006 5 Article10 16646864 10.2202/1544-6115.1209

[B33] Held GA Grinstein G Tu Y Modeling of DNA microarray data by using physical properties of hybridization Proc Natl Acad Sci USA 2003 100 13 7575 7580 10.1073/pnas.0832500100 12808153 PMC164628

[B34] Matveeva OV Shabalina SA Nemtsov VA Tsodikov AD Gesteland RF Atkins JF Thermodynamic calculations and statistical correlations for oligo-probes design Nucleic Acids Res 2003 31 14 4211 4217 10.1093/nar/gkg476 12853639 PMC167637

[B35] Benjamini Y Hochberg Y Controlling the False Discovery Rate - a Practical and Powerful Approach to Multiple Testing Journal of the Royal Statistical Society Series B-Methodological 1995 57 1 289 300

[B36] Storey JD A direct approach to false discovery rates Journal of the Royal Statistical Society Series B-Statistical Methodology 2002 64 479 498 10.1111/1467-9868.00346

[B37] Giles PJ Kipling D Normality of oligonucleotide microarray data and implications for parametric statistical analyses Bioinformatics 2003 19 17 2254 2262 10.1093/bioinformatics/btg311 14630654

[B38] Huang S Qu Y The loss in power when the test of differential expression is performed under a wrong scale J Comput Biol 2006 13 3 786 797 10.1089/cmb.2006.13.786 16706725

[B39] Sasaki D Kondo S Maeda N Gingeras TR Hasegawa Y Hayashizaki Y Characteristics of oligonucleotide tiling arrays measured by hybridizing full-length cDNA clones: causes of signal variation and false positive signals Genomics 2007 89 4 541 551 10.1016/j.ygeno.2006.12.013 17292583

[B40] Leiske DL Karimpour-Fard A Hume PS Fairbanks BD Gill RT A comparison of alternative 60-mer probe designs in an in-situ synthesized oligonucleotide microarray BMC Genomics 2006 7 72 10.1186/1471-2164-7-72 16595014 PMC1468409

[B41] Fan J Niu Y Selection and validation of normalization methods for c-DNA microarrays using within-array replications Bioinformatics 2007 23 18 2391 2398 10.1093/bioinformatics/btm361 17660210

[B42] Hughes TR Mao M Jones AR Burchard J Marton MJ Shannon KW Lefkowitz SM Ziman M Schelter JM Meyer MR Expression profiling using microarrays fabricated by an ink-jet oligonucleotide synthesizer Nat Biotechnol 2001 19 4 342 347 10.1038/86730 11283592

[B43] Naef F Lim DA Patil N Magnasco M DNA hybridization to mismatched templates: a chip study Phys Rev E Stat Nonlin Soft Matter Phys 2002 65 4 Pt 1 040902 12005798 10.1103/PhysRevE.65.040902

[B44] Hekstra D Taussig AR Magnasco M Naef F Absolute mRNA concentrations from sequence-specific calibration of oligonucleotide arrays Nucleic Acids Res 2003 31 7 1962 1968 10.1093/nar/gkg283 12655013 PMC152799

[B45] Levy JA Three new human herpesviruses (HHV6, 7, and 8) Lancet 1997 349 9051 558 563 10.1016/S0140-6736(97)80119-5 9048803

[B46] Jung SH Jang W How accurately can we control the FDR in analyzing microarray data? Bioinformatics 2006 22 14 1730 1736 10.1093/bioinformatics/btl161 16644791

