# Supplementary material for: Nonparametric methods for the analysis of single-color pathogen microarrays
Source: BMC Bioinformatics. 2010 Jun 28;11:354. doi: 10.1186/1471-2105-11-354 (PMC2909221; doi:10.1186/1471-2105-11-354)
Supplement: Additional File 3 — Figure S1. Graphical description of non-parametric tests evaluated in study [file 1471-2105-11-354-S3.PDF]

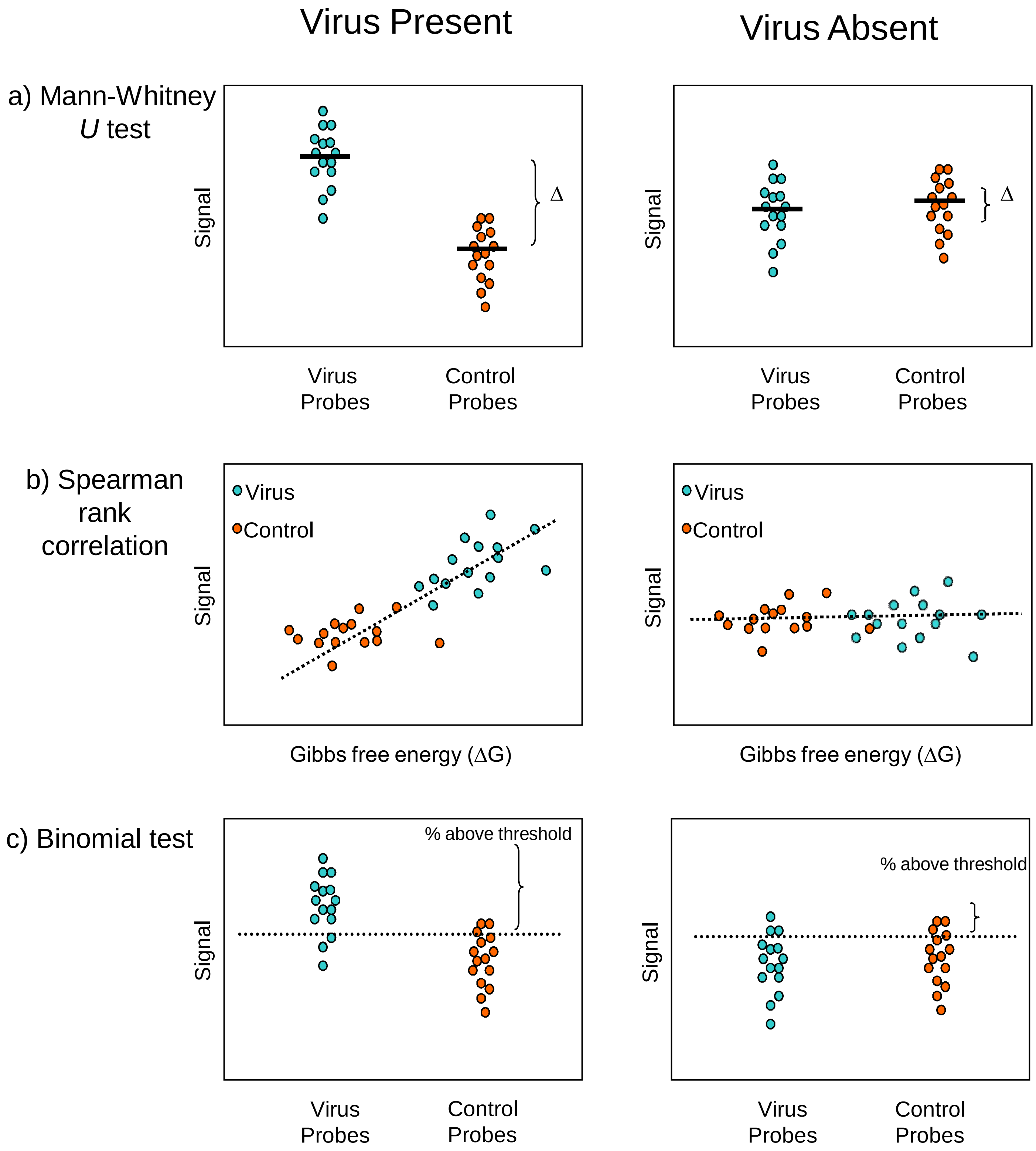

Figure S1: Non-parametric statistical tests

Illustrations of hypothetical probe signals when virus is present (left column) or absent (right column). (a) Mann-Whitney *U* statistic, tests the difference in central tendency; Δ represents difference in medians. Virus specific probes are predicted to have higher signal than control negative probes when virus is present. (b) Spearman rank correlation coefficient; tests the association of two variables. A high correlation between change in Gibbs free energy is expected when virus is present; when virus is absent, virus and control probe signal should be randomly distributed. (c) Binomial test, tests the probability of observing an event with two states, e.g., probes with signal above or below a threshold. When virus is present, a larger proportion of viral probes should be above threshold than control probes.
