# Supplementary material for: Nonparametric methods for the analysis of single-color pathogen microarrays
Source: BMC Bioinformatics. 2010 Jun 28;11:354. doi: 10.1186/1471-2105-11-354 (PMC2909221; doi:10.1186/1471-2105-11-354)
Supplement: Additional File 5 — Figure S2. Histograms of fluorescent signal for hybridizations to a pan-viral microarray. [file 1471-2105-11-354-S5.PDF]

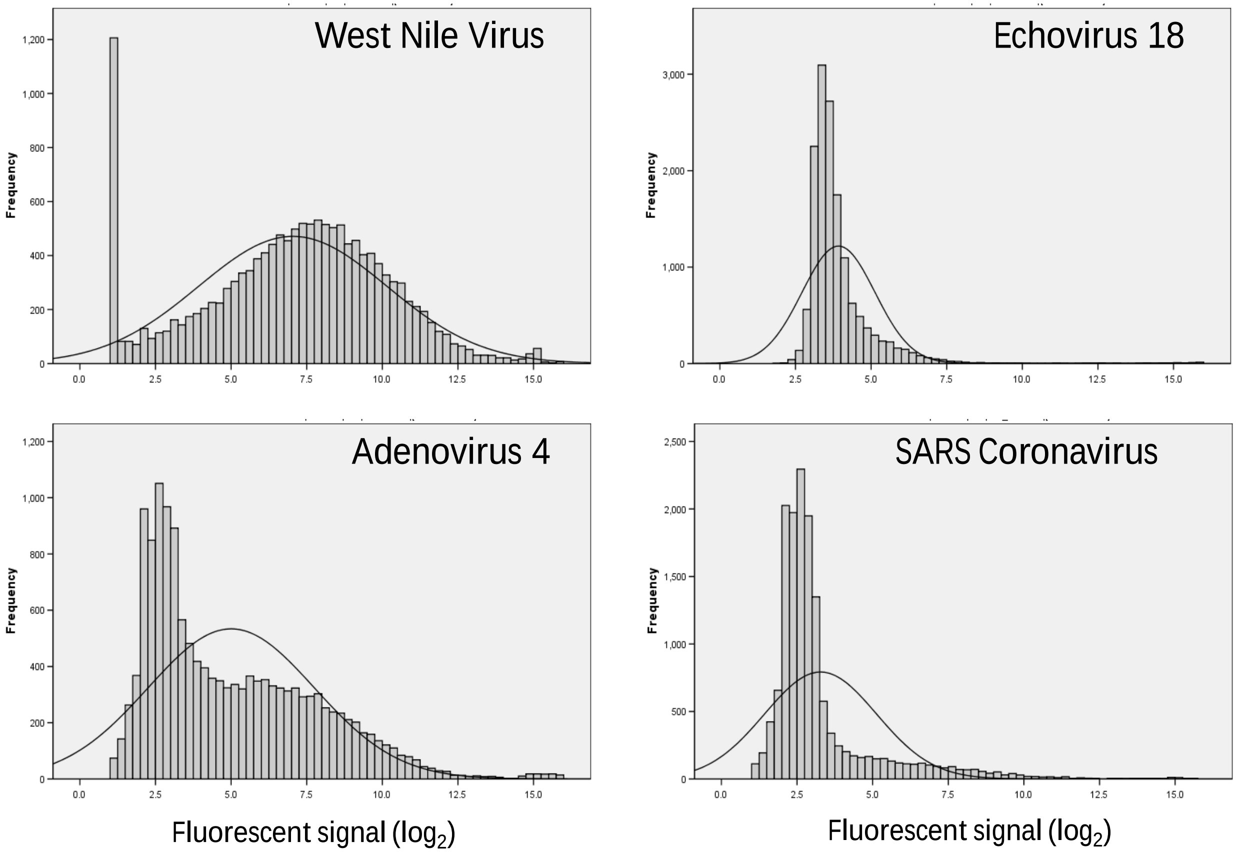

Supplementary Figure S2: Histograms of fluorescent signal for hybridizations to a pan-viral microarray
Nucleic acids extracted from viral isolates and hybridized to an array containing ~30,000 oligonucleotide probes. Fluorescent signal values were log transformed before plotting. Line shows frequencies for a normal (Gaussian) distribution.
