# Supplementary material for: Nonparametric methods for the analysis of single-color pathogen microarrays
Source: BMC Bioinformatics. 2010 Jun 28;11:354. doi: 10.1186/1471-2105-11-354 (PMC2909221; doi:10.1186/1471-2105-11-354)
Supplement: Additional File 6 — Figure S3. Comparison of probe printing strategies for pathogen arrays. [file 1471-2105-11-354-S6.PDF]

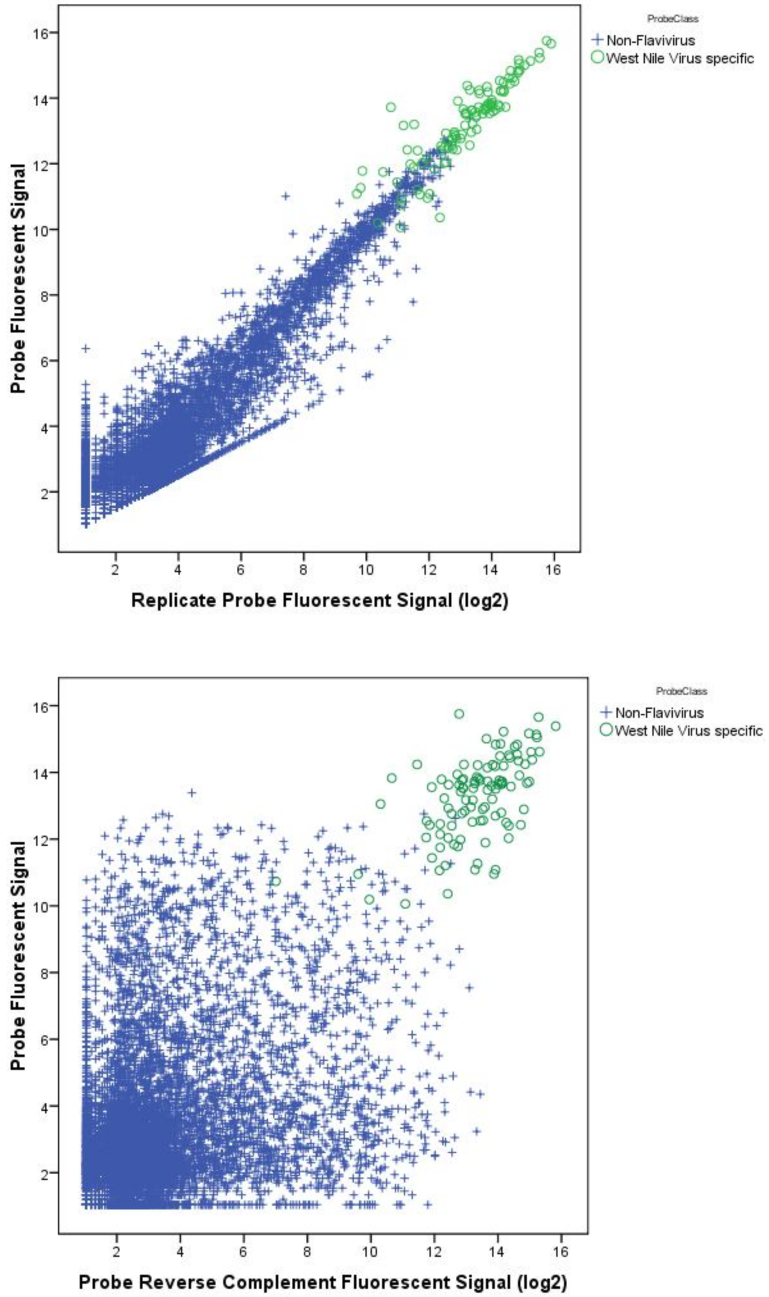

Figure S3: Comparison of probe printing strategies for pathogen arrays

West Nile Virus nucleic acid at 10^6 copies were hybridized to a pathogen array. Probes were printed in replicate and as reverse complements (genome anti-sense) in random positions on the microarray. Probes that are reactive to West Nile Virus are depicted as green circles, probes that are not reactive are depicted as blue crosses (e.g. they are probes for viruses outside of the flavivirus family). (A) Scatter-plot of fluorescent signal from pairs of replicate probes. (B) Fluorescent signal for probes and reverse complement pairs.
