# Supplementary material for: Nonparametric methods for the analysis of single-color pathogen microarrays
Source: BMC Bioinformatics. 2010 Jun 28;11:354. doi: 10.1186/1471-2105-11-354 (PMC2909221; doi:10.1186/1471-2105-11-354)
Supplement: Additional File 7 — Figure S4. Relationship between fluorescence and probe sequence composition. [file 1471-2105-11-354-S7.PDF]

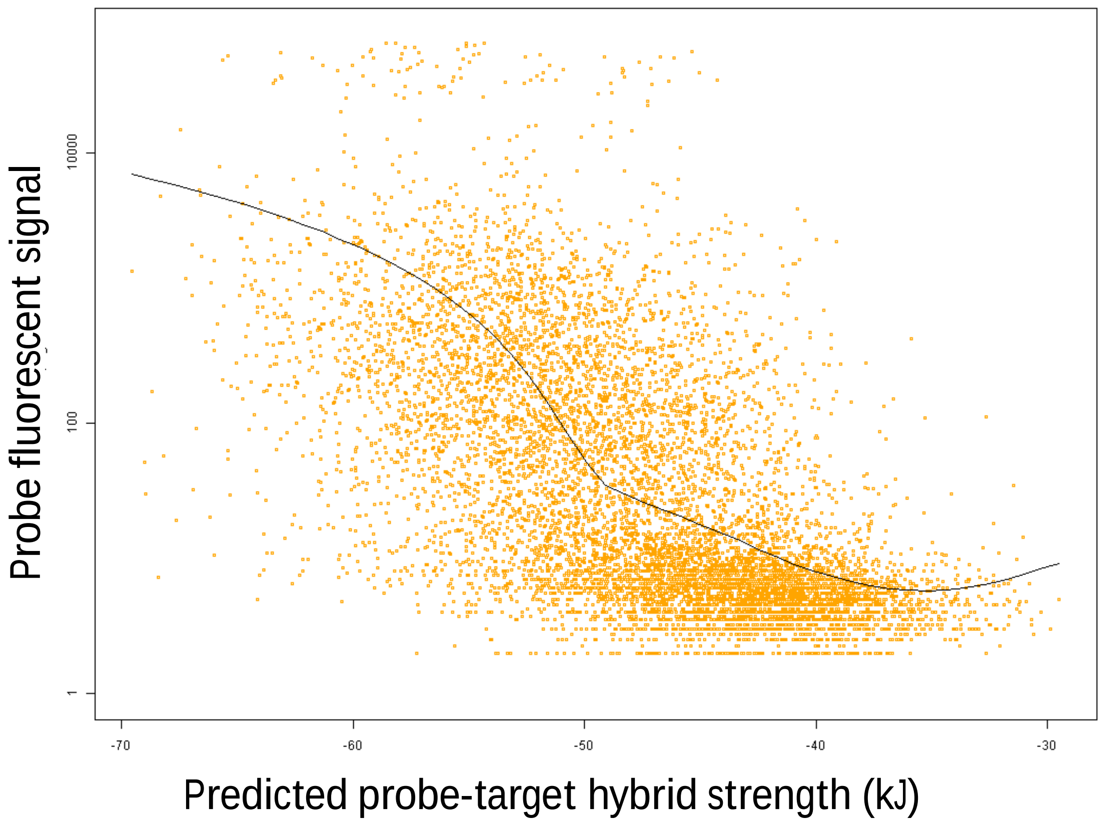

Supplementary Figure S4: Relationship between fluorescence and probe sequence composition
Human lung nucleic acid (200ng) was hybridized to a pathogen array. The strength of hybridization between a probe and its target sequence were computed (Gibbs free enegry, ΔG, x-axis) and plotted against fluorescent signal of viral-only responsive probes. A local fit was computed (Lowess fit, order 2, span 2) and plotted (black line).
