# Supplementary material for: Nonparametric methods for the analysis of single-color pathogen microarrays
Source: BMC Bioinformatics. 2010 Jun 28;11:354. doi: 10.1186/1471-2105-11-354 (PMC2909221; doi:10.1186/1471-2105-11-354)
Supplement: Additional File 10 — Table S1. Positive predictive value for methods of pathogen identification with no multiple testing correction. [file 1471-2105-11-354-S10.DOC]

| Table S1: Positive predictive value for methods of pathogen identification with no multiple testing correction | | | | | | | | | | |
| --- | --- | --- | --- | --- | --- | --- | --- | --- | --- | --- |
|  |  |  | |  |  |  |  |  |  |  |
|  | **Positive Predictive Value** | | | | | | | | | |
| **Target** | **Mann-Whitney *U*** | | | | **Spearman Correlation** | | | **Binomial test** | | |
|  | **sense** | **anti-sense** | **both** | | **sense** | **anti-sense** | **both** | **sense** | **anti-sense** | **both** |
| WNV | 71% | 65% | 77% | | 90% | 78% | 100% | 100% | 88% | 83% |
| HCoV-SARS | 11% | 25% | 20% | | 20% | 55% | 13% | 6% | 9% | 6% |
| EV18 | 90% | 93% | 87% | | 93% | 100% | 93% | 94% | 98% | 91% |
| LASV | 15% | 20% | 43% | | 0% | 9% | 33% | 13% | 20% | 25% |
| ZEBOV | 20% | 33% | 43% | | 100% | 100% | 100% | 100% | 100% | 67% |
| VSV | 50% | 50% | 17% | | 100% | 100% | 67% | 100% | 100% | 40% |
| HAdV-4 | 36% | 52% | 28% | | 81% | 95% | 78% | 70% | 78% | 58% |
| FLUA H1N1 | 95% | 89% | 88% | | 100% | 97% | 91% | 97% | 84% | 96% |
| HSV-1 | 22% | 26% | 14% | | 0% | 50% | 25% | 41% | 30% | 18% |
| **Average** | **46%** | **50%** | **46%** | | **65%** | **76%** | **67%** | **69%** | **67%** | **54%** |
| **Correct top ranked predictions** | **8 / 9** | **9 / 9** | **9 / 9** | | **6 / 9** | **7 / 9** | **7 / 9** | **8 / 9** | **9 / 9** | **9 / 9** |
| Top 250 predictions; *p value* threshold = 0.01 | | |  | |  |  |  |  |  |  |
